# Vaccinated Yet Booster-Hesitant: Perspectives from Boosted, Non-Boosted, and Unvaccinated Individuals

**DOI:** 10.3390/vaccines11030550

**Published:** 2023-02-25

**Authors:** Cheryl Lin, Brooke Bier, Rungting Tu, John J. Paat, Pikuei Tu

**Affiliations:** 1 Policy and Organizational Management Program, Duke University, Durham, NC 27708, USA; 2 Department of Business Administration, Tunghai University, Taichung 40704, Taiwan; 3 Department of Medicine, Duke University, Durham, NC 27708, USA

**Keywords:** vaccine hesitancy, confidence, acceptance, attitudes, health behavior, SARS-CoV-2, qualitative studies, decision sciences, risk perception, public health, communication strategies, immunization

## Abstract

Though available for all age groups in the US, only about half of those vaccinated have obtained a COVID-19 booster. Similar to the unvaccinated, those vaccinated-but-not-boosted may reduce the effectiveness of widespread viral protection. Booster hesitancy differs from general vaccine hesitancy yet remains less researched. We examined booster perceptions across vaccination status using qualitative methodologies. Four focus groups and 11 individual interviews (total n = 32) revealed nuanced changes and differences compared to the first-dose decision. Booster hesitancy stemmed from questions and surprises. Most vaccinated participants accepted the booster, though to varying degrees: enthusiastically with feelings of appreciation and added confidence, passively as an intuitive next step, indifferently following recommendation—“primed” by the yearly flu shot, and reluctantly with worries. The vaccinated-but-not-boosted group expressed confusion about the need for a new shot and discontentment as to why it was not communicated from the start, which coincided with their uncertainty about ending the pandemic. Inadvertently, booster recommendations further polarized non-vaccinated participants, augmenting their skepticism of the original dosages’ efficacy or necessity and intensifying their distrust of the government. The findings illuminate the need for adjusting vaccination promotions to better tailor communications (e.g., distinguishing its benefits from the first vaccine and emphasizing the continued risk of COVID-19 spread). Future researchers should further explore the vaccine-accepting-yet-booster-hesitant groups’ motivations and risk perceptions to reduce booster rejection.

## 1. Introduction

Over 265 million Americans are vaccinated with at least one dose against COVID-19, almost three years after the pandemic’s first reported US exposure [1]. Though cases are increasing at a slower rate than in prior years, COVID-19 still poses a threat to public health worldwide—some communities have experienced prolonged surges over the third winter, attributable to a combination of a persistent portion of the population objecting to vaccines [2], waning vaccination efficacy, and the continuing emergence globally of highly contagious variants [3,4,5,6]. The Food and Drug Administration (FDA) in September 2021 granted emergency use authorization for an adult booster [7] to augment viral protection for the population [8,9]. With clinical trials showing 95.6% efficacy against the Delta variant at the time, this additional shot was anticipated to be the next step toward ending the pandemic [10].

Researchers have extensively explored the reasons both for and against COVID-19 vaccine acceptance [11,12,13,14,15,16], as well as vaccine hesitancy through history. Vaccine hesitancy is defined as one’s delay of vaccination, though not always a complete rejection [17]. This phenomenon occurs universally and varies across nations and by data reporting practices [18]. Countries with higher education levels and access to healthcare services tend to have higher rates of vaccine hesitancy (e.g., Europe and Western Asia, compared to Southeast Asia) [19,20]. Factors of confidence, complacency, and convenience, as described by The World Health Organization’s 3C Model, are well understood to influence vaccine behavior [21,22]. Dating back to the 1800s, vaccine-hesitant individuals critiqued the “unnatural” nature of additional immune protection, such that vaccines expose an inactivated pathogen to a host’s immune system artificially [23,24]. These sentiments transpired further when false allegations of links between vaccine uptake and disease, such as the MMR vaccine and autism, degraded trust in vaccine safety [25]. In many countries, vaccine hesitancy is also a function of growing political polarization, where anti-vaccine rhetoric is associated with conservatism and distrust in science [26,27]. Thus, combating vaccine hesitancy remains a key initiative of public health officials, as reaching an adequate threshold of vaccination on a population level has the potential to eradicate viral spread [23,28].

Due to the booster’s relatively newer application, together with its assumed similarity to the primary shots, vaccine booster hesitancy remains less understood [29], with a dearth in particular of qualitative studies. Early findings on US individuals’ willingness to obtain a booster demonstrated that between 62% and 90% of previously-vaccinated individuals would accept a hypothetical booster [29,30,31,32,33,34], desiring additional protection against new variants or expressing worries that the current vaccination dose is no longer effective [3,31,34,35]. Conversely, some individuals expressed booster hesitancy, citing safety concerns with respect to either the vaccine itself or receiving multiple shots or feeling that a booster was unnecessary for protection [29,36,37]. Although previous COVID-19 vaccination history is the strongest predictor of whether a person decides to obtain a booster [37], as of the end of 2022, only 48% of vaccinated Americans have received a booster [1].

Few researchers have sought to capture the deeper sentiments behind booster hesitancy in vaccinated populations, which may be distinctly different from those of vaccine-rejecting individuals. The purpose of this study was to uncover the variations in opinions toward the booster and reasons for refusal. Through qualitative investigation, we aimed to explore the decision-making processes, the related feelings and uncertainty of individuals who received a primary COVID-19 vaccination but are hesitant about obtaining a booster shot, and how their attitudes differ from those who have accepted the booster without question as well as from the unvaccinated population. Raising confidence in and acceptance of the booster is an important part of decreasing the transmission of COVID-19 and helping to end the pandemic. This will be increasingly true as additional booster shots may be needed each year [38]. Our attempts to understand the attitudes and reactions of booster-reluctant individuals could furnish insights that will help inform interventions to better tailor campaigns for future booster rollouts and ensure continued protection against this virus.

## 2. Methods

### 2.1. Participant Screening and Recruitment

We conducted a qualitative study with both focus group (FG) and individual in-depth interviews (IDI) to assess participants’ perceptions in conjunction with their behavior related to the COVID-19 vaccination and booster shot. Individuals aged 18 or older living in the US were recruited through flyers posted at local clinics and retailers, social media groups (Facebook and Reddit), and personal network outreach (i.e., sending recruitment emails to friends and family and asking them to forward to people they know). We performed a two-stage screening: individuals expressed their interest in participating by completing a brief online questionnaire to provide information on their demographics, vaccine status, and contact information, along with preferred method and time to be contacted. Research team members then called potential participants to screen and schedule. Individuals who were unreachable after three attempts (email, text, voice mail, or a combination, depending on stated preference for correspondence) were excluded. Team members assessed respondents’ sound quality and intention in participation; individuals whose speaking or audio quality was difficult to comprehend, and those who seemed insincere or dishonest in responding to the simple screening questions (i.e., appeared to be primarily interested in receiving the incentive, their questionnaire and spoken responses did not match) were disqualified. Eligible participants were scheduled for a FG session according to their vaccination status. If they were not available on the designated date and time or preferred an individual interview, they were offered to choose a time for an IDI. We gave each participant an Amazon gift card to express our gratitude after each session.

To ensure we included individuals with different vaccination statuses and of different racial backgrounds, we selected a purposeful sample [39]. We planned for a balance between boosted and non-boosted participants and intentionally oversampled racial minorities to hear their voices since they are often under-represented in studies. Based on qualitative research guidelines and past studies, the sample size was initially projected to be 20–24 participants [40,41], including three FGs of different vaccination statuses. Transcriptions were reviewed on a rolling basis to observe emerging themes and evaluate content saturation. “Coding saturation” was achieved and recurring themes were identified before reaching 20 participants. We further invited more participants for FG and IDI to verify and contextualize the distinctive themes, attain “meaning saturation”, and fully capture the thematic issues and sentiments regarding the booster [42,43]. In total, we interviewed 32 individuals: four FGs of 4–6 participants each and 11 IDIs.

### 2.2. Interview Protocol and Strategy

We constructed a semi-structured discussion guide for both the FGs and the IDIs, tailored to the subgroups’ vaccination histories. All sessions explored general vaccine perceptions, COVID-19 vaccination and booster attitudes and decision-making, vaccine-related personal and social factors, sources of information searched or consulted concerning the COVID-19 vaccine and the interpretations drawn from those sources, and experience of or with the healthcare system.

An experienced moderator from a market research agency facilitated the FGs, and two researchers conducted each IDI. All sessions took place between February 2022 and April 2022, approximately three to five months after the FDA granted emergency use of the booster for all adults [7]. Both FGs and IDIs were conducted via Zoom (except for one FG, which was held in-person in North Carolina), which allowed us to recruit participants from around the US. Each FG lasted 90 min, and each IDI lasted an hour.

Informed consent was obtained from participants prior to each session. The research protocol was approved by the Institutional Review Board at Duke University.

### 2.3. Data Analysis

Group and individual interviews were recorded via Zoom, and each was transcribed and quality-checked by two researchers to ensure accuracy and completeness. All identifying information was removed from the transcripts before uploading text data into NVivo-12 (a qualitative analysis software by QSR International Pty Ltd., Burlington, MA, USA) [44] for coding and analysis. Data was stored in Duke University’s secured cloud-based storage Box.

Two researchers devised a draft codebook, and together the research team made several revisions by test-coding sample transcripts and group discussion. We employed an inductive and thematic approach to identify and refine emerging themes [45], which was first conceptualized by identifying commonly mentioned points or sentiments across sessions and substantiated by reviewing and corroborating all transcripts. We selected quotes to represent each theme, and the quotes were grouped to convey larger trends in participant sentiment.

## 3. Results

A total of 32 participants took part in either FGs or IDIs, with diverse demographics and vaccination statuses (Table 1 and Table 2). Their perspectives on the COVID-19 vaccine and booster differed widely, ranging from very receptive to surprised, worried, or doubtful along the spectrum of vaccination status. We grouped these attitudes and presented them accordingly, illuminating the intensity of sentiments and the unexpected consequences of the perceptions of and trust in vaccines more generally. Responses within subgroups were similar in content across the two interview formats, with no apparent thematic differences aside from personal anecdotes, and thus were grouped interchangeably in the following reporting.

### 3.1. Booster Acceptance & Motivations

Among vaccinated participants, attitudes and behavior toward the booster varied. Less than half of them had enthusiastically obtained or were planning to obtain their booster shot. They embraced the booster as “the best thing” to diminish the continuing pandemic and trusted the vaccine’s efficacy in preventing the spread of the virus. These participants were among the first to get boosted once the shot was available in their communities or for their age group.

*“I feel like the booster is literally like (hand motion of blowing up) … we actually get a little dose of superpowers, and we’re able to survive our enemy for a little bit longer.”*—Participant #1

*“When I heard about the third shot, I said, okay, I want to go ahead and do it.”*—#5

*“I was very grateful… I want to be under the most protection I can be to be with them [my parents] and not to make them sick.”*—#10

Others accepted the booster more passively as it appeared to be the intuitive next step to further protecting themselves, as well as an extra push toward “returning to normal”. These participants generally had positive experiences with the first two doses, so they felt their decision to get the additional shot was relatively easy.

*“I already got two, so I was like [shrugs shoulders].”*—FG1-#2

*“Like, oh, I guess I gotta do this right now, ‘cause why did I already did all of the other ones? So why not just finish it up.”*—FG1-#5

Some participants seemed indifferent toward getting the booster, yet they still accepted it, tolerating the nuisance of repeating an inoculation. They acknowledged that multiple doses were needed, similar to an annual flu shot.

*“I feel like I have been in a way, primed to be okay with getting yearly vaccinations, just because growing up, my parents always took me to my flu shot appointment.”*—#11

*“It’s sort of like cousins of common cold, the regular flu that you know, an annual booster shot was like… just made sense to me from the get-go.”*—#7

Others had questions and reported their concerns or uncertainty, accepting the booster more reluctantly and feeling unsettled about possibly getting a new shot each year.

*“I wonder if we have to take this every year now, like the flu vaccine. Are we going to have to do this every year ….”*—FG1-#1

*“I’m waiting to see whether there’s something else [side effects] that may come up, then if there’s nothing, then I’ll just take the booster.”*—FG2-#3

### 3.2. Attitudes Surrounding Booster Hesitancy and Rejection

A prominent concern surrounding booster hesitancy was a lack of understanding as to why a third dose was needed. For non-boosted individuals, differences in perceptions were observed between the vaccinated and the non-vaccinated. The former were not necessarily resisting a booster but were surprised that the first one or two shots were not sufficient; many were confused about or displeased concerning the fact that the booster’s necessity had not been stated from the start of the COVID-19 vaccine rollout.

*“I was like, okay, is this really necessary? Was this not good enough at first?”*—#2

*“I understand that it [the booster] can definitely be helpful, but, after a while getting the same exact shots, it’s going to be... I don’t know how efficient that can be.”*—#9

*“I don’t think it was as important as the initial round of vaccination.”*—#4

One subset of vaccinated-but-not-boosted individuals felt receptive to the booster but had not obtained it as of the date of their FG or IDI. Logistical or health reasons prevented them from going forward with the booster; they also showed that some encouragement would convince them to take the additional shot.

*“I was going to get it, but then I got COVID that same night, so I have to wait 90 days until I’m able to get it.”*—FG4-#5

*“Other things in life just, just popped into my head… I think after this conversation with everybody, I’m going to go right down on my calendar to make arrangements to get this done.”*—FG2-#4

The hesitancies of a few vaccinated individuals who nevertheless rejected a booster were based on their adverse reactions to the first or second dose, which provided enough disincentive to not take the booster. They mentioned various side effects of the vaccine as well as new health conditions after getting the vaccine (though the link may or may not have been clinically identified). They affirmed that they did not want to further risk their well-being by getting the booster.

*“I have personal concerns because I was affected by the original vaccinations, I had a health issue, and then I just thought about it because it did kind of come on two months after I was fully vaccinated.”*—#4

#### Consequences of the Booster—Increased Distrust

The non-vaccinated and non-boosted (or not intending to get boosted) participants reported more concern about the initial vaccine rather than confusion, fear, or logistical issues about the booster. These participants were skeptical of the speed at which the vaccine was developed or approved, its unknown long-term effects, and the fact that the booster had required even less time than the original vaccine to be developed and recommended.

*“Why are we forced to do both of them if it [the vaccine] is good?”*—FC3-#5

*“I think if they had taken the time to develop something, we would just need the vaccine once, and we’d be comfortable with it.”*—#6

These participants expressed a sense of distrust in the vaccine or the healthcare system and government, and they took the booster recommendations as evidence that the vaccine did not work or something was not quite right.

*“I think the booster is just a way of trying to convince us that the COVID-19 vaccine is actually working, which isn’t true.”*—FG3-#2

*“They thought, well, I’m covered, you know. So, when they came out later on and are like, ‘no, you get to get another booster,’ and I think that just was… a little fishy.”*—#3

*“I think probably it [the vaccine] is water, drip water or something, because if it has to be topped up every time, then … the effectiveness of it is something that I can question.”*—#6

### 3.3. Booster’s Influence on Perceptions toward the Pandemic and Subsequent Shots

Some boosted participants expressed optimism about resuming their previous lives due to increased community protection, i.e., the booster was in fact effective because infection rates were declining. Consequently, they felt less urgency about obtaining a future fourth or fifth shot.

*“I feel like after getting a booster and then seeing life return, or like at least life on campus return relatively back to normal.”*—#11

*“When we got the booster, it was in the height of when the Omicron variant was really rampaging, and that was kind of a rough one. So, I think we were all a little bit more willing to get it because of the variant … I don’t feel as worried about that one. So that’s why I’m not as eager to get my fourth [shot].”*—#12

However, both the unvaccinated and those who were vaccinated-but-rejected-booster reported shared attitudes that the booster did not signal a return to normalcy, but rather the opposite. The more the need for a booster was promoted by the health authorities and discussed in the media, the more pessimism they felt about the end of the pandemic.

*“Anytime you hear booster, it just makes it feel like this is never going to end.”*—#3

*“I feel like basically, if one shot wasn’t enough to, like, I guess survive the pandemic, then it basically means that you’re gonna need more ‘cause they also don’t see it going anywhere.”*—FC3-#4

*“Everything is going to go back to normal. And then it’s like, oh, just kidding. We need a booster.”*—FC1-#2

**Figure 1:**
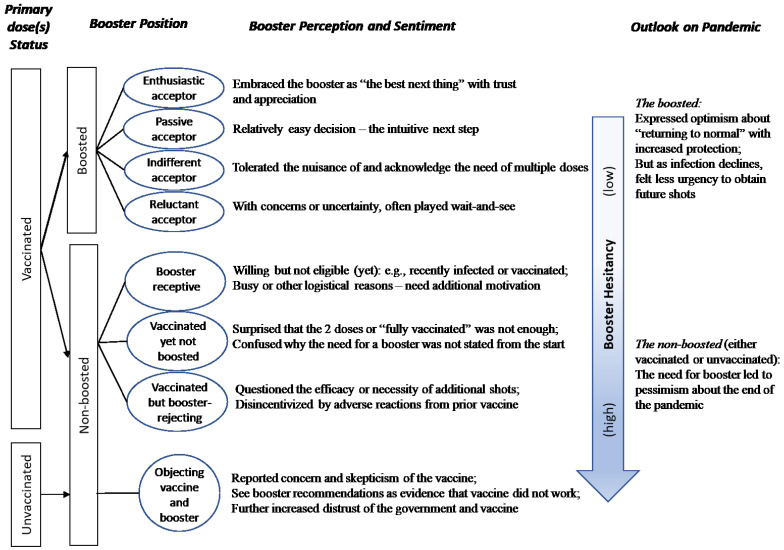
Participant perceptions and sentiments toward COVID-19 booster grouped by vaccination status and level of booster hesitancy.

Figure 1 presents a visual depiction of the key findings grouped by participants’ vaccination status and along the continuum of low to high booster hesitancy.

## 4. Discussion

Our qualitative inquiry distinguished themes on perceptions of the COVID-19 booster relative to individuals’ vaccine uptake, ranging from added trust and appreciation to worry or increased skepticism. The findings revealed both shared similar as well as dissimilar sentiments across the vaccinated-and-boosted, the vaccinated-but-not-boosted, and the unvaccinated groups. Uncertainties surrounding the booster, even within vaccine-accepting groups, uncovered differences in decisions and motivations for obtaining the original vaccine and obtaining a booster. Booster recommendations further solidified some participants’ stances toward the original vaccine (i.e., that it worked or did not work).

We uniquely illuminated the nuances of participant attitudes and behavior toward the booster, stratifying four subgroups of acceptors with varied positions—enthusiastic, passive, indifferent, and reluctant, portrayed with their own narratives. Our results provide deeper insight into the rationale behind booster acceptance, including the hesitancy by those boosted and those vaccinated-yet-booster-resisting, which have not been explored previously. For instance, the reasoning of enthusiastic acceptors mirrored those reported earlier for the boosted in general, such as a desire for extra protection against new variants or their trust in the vaccine’s efficacy [3,31,34,35,46]; however, these motivations did not apply to all boosted participants in our study. Indifferent acceptors were especially attuned to the fact that a (periodic) booster might be needed for years to come. Accepting the COVID-19 vaccine on the same logic as that employed in the acceptance of an annual flu vaccine aligns with existing reports, in which vaccination history predicted booster receptivity: individuals vaccinated against influenza displayed more willingness to obtain a COVID-19 booster, a trend also found for the acceptance of either the initial COVID-19 vaccine or the H1N1 vaccine [29,36]. Our results provide further evidence of this relationship, suggesting that framing the booster as a yearly healthy habit, like the flu shot, may mitigate repeated decision contemplation and improve uptake. Nevertheless, given that just slightly over half of the US population is vaccinated against influenza [47], this strategy may only be effective for those already primed for and accepting of an annual flu shot.

Among the non-boosted, whether vaccinated or not, sentiments of resistance often stemmed from their skepticism, fear, or perception of a threat. Key themes associated with objections to a COVID-19 booster included the idea that an additional shot was unnecessary or unsafe—common reasons also reported by those who rejected the original vaccine [29,36] and similar to those indicated in recent papers on booster-refusing populations [29,34,37]. Of particular note, our qualitative analyses uncovered the augmented distrust of the vaccine attributed to booster recommendations. For those participants who already doubted the original vaccine’s safety or efficacy, the introduction of the booster reinforced their suspicion concerning whether the initial shot was warranted or effective, especially since the two-shot regimen was considered “fully vaccinated” [48]. Perceiving the booster as unnecessary was echoed by individuals who accepted the initial vaccine but rejected the booster, who were more aligned with unvaccinated than vaccinated individuals in their views of the booster and its role in preventing or reducing transmission of the virus. However, it appeared that these participants were more confused by the need for a booster than skeptical of it, primarily because they had assumed they were well protected by the initial vaccine. This sentiment is reflective of the complacency construct in the 3C Model, where one views vaccination as unnecessary given a low perceived risk of infection [22].

Many participants expressed surprise and discontentment that the need for more shots was not stated from the start. The resulting unsettledness drew questions about the introduction of the original vaccines as well as the booster. This indicates that communications around the booster should be clearer in explaining the evolving pandemic and continued risk. Thus, health authorities should reconsider their strategies for presenting and promoting the booster, both current and future versions, so that hesitancy is reduced and erosion of trust is prevented. Further, the booster-questioning subgroup would benefit from educational communication showcasing the booster as an enhancement to strengthen the protection of the vaccine over time.

### Limitations and Future Research

The study had several limitations. Group dynamics within the FGs could promote conformity among participants due to peer pressure; the moderator encouraged opinions that offered a new perspective from the majority or the previously voiced comment. To complement this, the IDIs allowed individuals to share their experiences more freely. In analysis, the process of developing themes and choosing quotes may have introduced confirmation bias. We were mindful of minimizing this bias, including employing multiple text coders, jointly refining themes, and collectively selecting the most representative quotes.

Our purposive sampling was intended to include views from all vaccine positions to investigate the diversity in behavior and perspective. While we achieved a balance in vaccination status (ranging from completely unvaccinated to vaccinated-and-boosted) and a few demographic variables, we were unable to concurrently control other ratios. There was a higher proportion of participants with a household income of $50,00–99,999 and $100,000–199,999, while white respondents were much under-sampled, potentially making the results less representative. Nevertheless, we observed differences in perceptions across vaccination status but not by demographic factors. Further, our volunteer-based and non-anonymity of FG and IDI excluded participants who were not aware of our recruitment or were less willing to participate face-to-face (even through the internet). Thus, our findings may have missed some perspectives. The timing of the investigation played a role in the reported views as well. Opinions may have shifted due to the pandemic’s continued evolution, as well as before and after the FDA granted emergency use of the booster.

As booster approval for all age groups is relatively recent, more research is needed. For example, a gap in the literature exists comparing vaccine intention to action. This issue is critical in light of reported high levels of booster willingness but lower actual uptake [49]. Our qualitative inquiry indicated two possible explanations for this discrepancy: some individuals have recently gotten COVID-19 and thus were ineligible for the booster for a few months, or they deemed their infection as effective natural immunity. Future researchers could investigate incentives to turn such individuals’ interest, intention, or fear into increased booster uptake, as well as how booster mandates impact vaccine attitudes and decisions. Additionally, previous scholars have identified strategies to overcome barriers to action for COVID-19 vaccination, such as text reminders, locating testing sites in convenient settings (e.g., the workplace), or using motivational language [50,51]. Future campaigns can tap these conceptual interventions, which may prompt individuals to remember their initial motivation for getting vaccinated.

## 5. Conclusions

Understanding perceptions of the COVID-19 booster shot is extremely relevant, yet qualitative research is lacking regarding deeper public booster experience and opinion concerning acceptance. The vaccinated-but-booster-hesitant population remains a unique target for intervention, as we demonstrated that vaccinated individuals might not necessarily be receptive to or understand the need for additional shots. Moreover, it is important that the same intensity and scope used in the original vaccine’s push be used to promote the booster, especially given public health authorities’ expectations of future booster iterations required for emerging variants. However, strategies employed to convert anti-vaccine groups may be less effective in increasing booster acceptance due to differences in perceptions between the booster and first dose as well as motivations behind behavior change. Our findings further emphasize that campaigns would benefit from clearly explaining why a booster is required, framing the necessity in a similar fashion to the annual flu shot, and accurately presenting the booster to be intended to reduce the impact of otherwise severe symptoms or mortality rather than preventing disease. If the booster was thus framed, the public would be less prone to misinterpreting the initial vaccine or the booster shot as non-effective and therefore questioning or rejecting the recommendation.

## Figures and Tables

**Table 1:** Participant demographics (n = 32).

Demographic Variable		n (%) *
Age	18–22	4 (12.5%)
	23–29	8 (25.0%)
	30–39	10 (31.3%)
	40–49	4 (12.5%)
	50–59	6 (18.8%)
Gender	Female	17 (53.1%)
	Male	14 (43.8%)
	Prefer not to answer	1 (3.1%)
Race	African American	13 (40.6%)
	Hispanic	9 (28.1%)
	Asian	5 (15.6%)
	White	4 (12.5%)
	Native American	1 (3.1%)
Household Income (US$)	<$25,000	1 (3.1%)
	$25,000–49,999	7 (21.9%)
	$50,000–99,999	9 (28.1%)
	$100,000–$199,999	10 (31.2%)
	>$200,000	4 (12.5%)
	Prefer not to answer	1 (3.1%)

* Some categories may not add up to 100% due to rounding.

**Table 2:** Participant Vaccination Status (n = 32).

Vaccination Status	FG n = 21	IDI n = 11	Total n = 32
Fully vaccinated and received at least one booster	9 (42.9%) *	6 (54.5%)	15 (46.9%)
Vaccinated, non-boosted	6 (28.6%)	3 (27.3%)	9 (28.1%)
Not vaccinated (non-boosted)	6 (28.6%)	2 (18.2%)	8 (25.0%)

* Some categories may not add up to 100% due to rounding.

## Data Availability

The deidentified data related to this paper is available from the corresponding author one year from the date of publication upon reasonable request with methodologically sound research proposal.
